# Poisonous Oysters

**Published:** 1883-11

**Authors:** 


### POISONOUS OYSTERS.

During the unseasonably warm weather, which occurred at intervals in the last two months, we saw four or five cases of apparent poisoning from eating oysters in this city. The sufferers were disposed to attribute it to some infected or diseased condition of the oysters, unusual and not to be anticipated in months whose names contain the letter *r*. October and November both contain the traditional letter.

This gives us an opportunity to correct the popular error in regard to oysters and the letter *r*. Let it not be forgotten that oysters are dangerous food, no matter what month they may be eaten in, if the prevailing weather be warm. A rigid observance of this fact will save many a doctor's bill and gastric gripe.

